# Pathological interactions between the endothelin-1 and the angiotensin- converting enzyme among Tunisian coronary patients

**DOI:** 10.1186/s12872-016-0417-x

**Published:** 2016-11-29

**Authors:** Abdelkader Chalghoum, Yosri Noichri, Azza Dandana, Bruno Baudin, Abdelhédi Miled, Salima Ferchichi

**Affiliations:** 1 Laboratory of Biochemistry, Farhat HACHED Hospital, Street Doctor Moreau, 4000 Sousse, Tunisia; 2 Valorization and Technology Transfer Space, Center of Biotechnology of Borj Cedria, 2050 HamamLif, Tunisia; 3 Department of Biochemistry, Saint-Antoine Hospital, 184 Street Faubourg Saint-Antoine, 75571 Paris, Cedex 12 France

**Keywords:** Acute coronary syndrome, Endothelin-1, Angiotensin-converting enzyme, Risk factors, Metabolic interactions

## Abstract

**Background:**

The correct understanding of the biochemical and metabolic interactions between coronary risk factors contribute to the exploration of cardiovascular pathophysiology and improves therapeutic care.

The aim of this study was to explore the endothelin-1 (ET-1) concentration and the angiotensin converting enzyme (ACE) activity among Tunisian patients with coronary heart disease, and to investigate the metabolic relationships between these two markers,… and to assess the possible relationship between them and the different risk factors.

In this present study, ET-1 concentration was determined by an analytical method (High Performance Chromatography, coupled by Mass Spectrometry), ACE activity was measured by a kinetic method for patients and healthy controls. These subjects (157 patients and 142 controls) beneficed also by a biochemical exploration (lipid, apolipoproteins and glucose profiles) to quantify cardiovascular risk.

**Results:**

A statistically significant increase of the ET-1 concentration was found among patients compared to healthy controls (15.2 ± 5.3 nM vs 7.1 ± 2.7 nM, p < 0,00001). For the ACE activity, in spite the treatment of the majority of patients (97%) with ACE inhibitors, this activity was statistically elevated in patients compared to healthy subjects (86.7 ± 25.4 IU/L vs 42.8 ± 12.1 IU/L, *p* < 0.00001).

Furthermore, a statistically positive correlation was identified between these two cardiac markers (*r* = 0.68 *p* < 0.00001).

**Conclusion:**

The study of the metabolic relationship between the ET-1 and ACE among coronary patients reveals other therapeutics targets.

## Background

Acute Coronary Syndrome (ACS) is classified among the major cause of mortality and morbidity in the western countries. The diagnosis of this ischemic syndrome is essentially based on clinical examination (electrocardiogram), the biological examination (serum level of cardiac troponin) is used to identify the necrotic damage. This disease is multifactorial, complex and polygenic. The risk factors are varied and diversified, according to the Framingham reference study; these factors are classified into modifiable factors (smoking, arterial hypertension, diabetes, obesity …) and un-modifiable factors (age, gender, heredity…). Atherosclerosis is the main etiology of this syndrome [1–4].

However, the endothelin-1 (ET-1) and the hyperactivity of the angiotensin- converting enzyme (ACE) are classified among the coronary “unconventional risk factors” [5–7].

ET-1 is an endothelium derived, potent vasoconstrictor peptide of 21 amino acids (2.5 kDa with a great structural homology with snake venom, the S6 sarafotoxin). It was finally isolated and sequenced from endothelial cell culture by Yanagisiwa M. in 1988. Its peptide is strongly implicated in the ACS’s genesis and complications by its vasoconstrictors, pro-oxidant and thrombotic effects [7–9]^.^

ACE (peptidyl-dipeptidase with zinc, EC, 3,4,15,1) is well known by its physiological role in the rennin angiotensin system by its role in angiotensin-I cleaving (an inactive decapeptide) to generate angiotensin II (a powerful vasoconstrictor) and its role in bradykinin hydrolysis (a potent vasodilatator). This enzyme stimulates also the release of aldosterone from the adrenal cortex, leading to sodium retention [10–12].

In this context, comes the aim of our study which is to quantify the serum concentrations of ET-1 and to measure the ECA serum activity in Tunisians coronary compared to controls subjects, investigate the impact of various risk factors on serum variation of these two parameters as well as the study of the correlation between these two vasoconstrictors.

## Methods

### Populations study

This is a prospective study, in which sampling was carried between January 2010 and November 2011. One hundred fifty seven Tunisian coronary patients (121 men and 36 women) middle-aged (64.8 ± 11.7 years) were recruited from the Cardiology Service of Farhat Hached, Hospital of Sousse, Tunisia. One hundred forty two healthy subjects (111 men and 31 women) middle-aged (56.8 ± 9.4 years) were considered as the control group. This age difference is unavoidable, since at a more advanced age, the risk factors accumulate, and the possibility to find healthy subjects non carriers of any risk factor becomes almost impossible (since the advanced age it is even a risk factor). So we were here between two choices: either having patients and "healthy subjects" holders of risk factors with a close age, or having a remarkable difference (not significant) of age and healthy controls without risk factors.

The patients and healthy subjects signed a free and clear consent which explains the objectives of this work with an undertaking not to publish the names of participants, their personal data including test results (following the instructions of Tunisian National Committee of Medical Ethics, consistent with the Declaration of Helsinki).

The approval of compliance with the Helsinki declaration was signed in December 2009. A datasheet had been prepared for each subject (patient or control) to identify cardiovascular risk factors and to know the susceptibility degrees to ACS. This sheet contains the anthropometric characteristics, the biological parameters, the risk factors, the treatments of patients, the exclusion factors (thyroid diseases, sarcoidosis, Gaucher disease).

### Laboratory analysis

Venous blood samples were drawn after 12 h overnight fast. A collection of two tubes were made for each patient and witness: one tube without anticoagulant for determination lipid parameters, ET-1 concentration, and ACE activity and, the second one is heparinized tube for blood glucose. Serum total cholesterol (TC), triglycerides (TG), high density lipoprotein cholesterol (HDL-C) and glucose were measured with colorimetric essay using an automated system (Cx9 Pro-Beckman Coulter-Fuller-Ton CA). Low density lipoprotein cholesterol (LDL-C) was determined by Friedewald formula for TG levels below 4.5 mmol/L. Apolipoproteins (ApoA1 and Apo B) were determined by immunonephelometry (Cobas Integra 400, Roche).

After plasmatic ET-1 extraction with ethanol, the concentration was measured by High Performance Chromatography, coupled to Mass Spectrometry (3200 Q TRAP LC/MS/MS system),according the Walczak M protocol, 2010 [9],using a synthetic standard (ET-1 Sigma-Aldrich St. Louis, MO, USA) for the specific spectra identification and for the calibration curve determining. Chromatographic separation was carried out with C18 analytical column (30 mm x 2.1 mm, 3.5 μm, Waters Ireland) set at 20 °C. Two solvents mixtures were used: solvent A: Acetonitrile and solvent B: H_2_O. The following gradient was used: 0–5 min 0–100% A; 5–7 min 100% A; 7–8, 100–0% A; 8–15 min, 100% B. The flow rate was set at 300 μlmin^−1^ and a sample volume of 25 μl was injected in the analytical column. The linearity zone (1.6 nM - 160 nM) is established after calibrationby a purified bovine serum (with 3 repetitions for each concentration)

ACE activity was determined by kinetic method at 340 nm, according the Chalghoum A protocol, 2012 [4], using a synthetic substrate, Furylacryloyl-phenylalanyl– glycyl-glycine (FAPGG) (Trinity Biotech,St Louis USA), with an initial measurement at t = 0 min and a final measurement at t = 5 min.

### Statistical analysis

Database management and statistical analyseswere carried out using SPSS (**S**tatistical **P**ackage forthe **S**ociological **S**ciences), version 21.0 (IBM). Results are presented as means ± SD, or percentages. Means were compared using Student test. The relations between variables were assessed with Pearson’s correlation analysis.

These parametric tests (means comparison, statistical correlations) were performed under of the analytical statistics conditions (Gaussian dispersion for the different parameters, *n* ≥ 30 for each group and subgroup…). The significance threshold was set at 5% (*p* < 0.05).

## Results

Table 1 summarizes the anthropometric and clinical data of patients and health subjects. Hypertension, diabetes, obesity, tobacco, heredity… are the majors risk factors. However our control subjects are not exposed to these factors

**Table 1:** Anthropometric characteristics and clinical data of patients and control subjects

	Patients (*n* = 157)	Control subjects (*n* = 142)
Age (x ± σ years)	64.8 ± 11.7	56.8 ± 9.4
Sex		
Men (%)	121 (77%)	111 (78.2%)
Women (%)	36 (23%)	31 (21.8%)
BMI (kg /m^2^)	27.6 ± 4	23.3 ± 2.2
Hypertension (%)	88	0
Obesity (%)	52	8.5%
Diabetes (%)	64	0
Smoking (%)	62.4	7
Family cardiachistory (%)	68	6
Personnel cardiachistory (%)	66	0
Postmenopausal women (%)	100	82
Dyslipidemia (%)	40	0
Sedentary (%)	43	11
Alcohol (%)	33	14
Treatment : ACE inhibitors (%)	97	0
Statins (%)	38	0
Beta-Blockers (%)	33	
Ca-Blockers (%)	27	0
Diuretics (%)	17	0

*X* mean

*σ* standard deviation

*BMI* Body mass index

Biological parameters and the values of ET-1 and ACE in patients and controls subjects are shown in Table 2.

**Table 2:** The distribution of biological markers, ET-1 concentration and ACE activity in patients and control subjects

Populations Biological parameters	Patients (*n* = 157)	Control subjects (*n* = 142)	*p*
*Glucose (x ± σmmolL/)*	9.8 ± 4.2	5.40 ± 0.84	<0000.1
*TC (x ± σmmol/L)*	5.70 ± 3.1	4.60 ± 2.6	<0.00
*HDL-C (x ± σmmol/L)*	1.14 ± 0.22	1.30 ± 0.41	NS
*LDL-C (x ± σmmol/L)*	3.60 ± 2.16	2.80 ± 1.4	<0.001
*TG (x ± σmmol/L)*	1.60 ± 0.9	1.24 ± 0.3	NS
*ApoA1 (x ± σ. g/L)*	1.41 ± 0.62	1.80 ± 0.2	<0000.1
*ApoB (x ± σ. g/L)*	1.40 ± 0.81	0.70 ± 0.2	<0000.1
*ET-1 (x ± σ. nM)*	15.2 ± 5.3	7.1 ± 2.7	<0.00001
*ACE (x ± σ. UI/L)*	86.7 ± 25.4	42.8 ± 12.1	<0.00001

*NS* not significant (*p* > 0.05)

*X* mean

*σ* standard deviation

*TC* Total cholesterol

*HDL-C* high density lipoprotein cholesterl

*LDL-C* low density lipoprotein cholesterol

*TG* triglycerides

*ApoA* Apolipoprotein A

*ApoB* Apolipoprotein B

*ET-1* Endothelin-1

*ACE* Angiotensin-converting enzyme

Glucose, TC, LDL-C,and apolipoprotein B(Apo B) were significantly increased in patients compared to controls. Unlike apolipoprotein A1 concentration was significantly higher among control group compared to patients. HDL-C, and TG showed no significant difference between two populations.

**Fig. 1:**
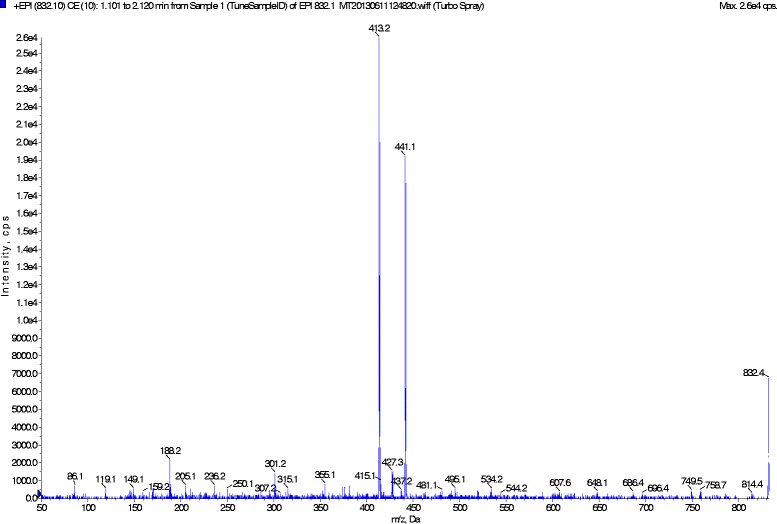
The mass spectra presentation of the ET-1 peptide, triply ionized, with a peak of 832.4 Da (M / Z)

Figure 1 illustrates the mass spectra of the ET-1 peptide, triply ionized, 832.4 Da (M / Z).

In our study, a statistically significant elevation of the ET-1 concentration was observed among patients compared to healthy subjects. For the ACE activity, although the majority of our patients (97%) were treated with ACE inhibitors, this activity was statistically elevated in patients compared to controls subjects.

Biochemical exploration showed that these two vasoconstrictors depend on gender, arterial hypertension, tobacco, obesity and dyslipidemia, unlike diabetes, Personal cardiovascular antecedents, alcohol intake and physical inactivity (Tables 3 and 4).

**Table 3:** The variation of the ET-1 concentration among patients according risk factors

Populations and risk factors	ET-1 (nM/L)		*p*
Gender	Men (n = 121)	17.4 ± 4.6	<0.00001
	Women (n = 36)	7.8 ± 2.3	
Hypertension	Yes (n = 138)	16.4 ± 3.3	<0.00001
	No (n = 19)	6.6 ± 1.9	
Diabetes	Yes (n = 101)	15 ± 5.1	NS
	No (n = 56)	15.6 ± 3.8	
Tobacco	Yes (n = 98)	11.6 ± 3	<0.00001
	No (n = 59)	21.2 ± 6.1	
Personal cardiovascular antecedents	Yes (n = 104)	15.3 ± 4.4	NS
	No (n = 53)	15 ± 2.8	
Obesity	Yes (n = 82)	12.2 ± 5.3	<0.001
	No (n = 75)	18.2 ± 4	
Dyslipidemia	Yes (n = 63)	11.8 ± 1.6	<0.00001
	No (n = 94)	17 .5 ± 4.6	
Alcohol	Yes (n = 52)	16 ± 4.1	NS
	No (n = 105)	14.8 ± 3.8	
Sedentarity	Yes (n = 67)	15.7 ± 3.7	NS
	No (n = 90)	14.9 ± 2.4	

*NS* not significant (*p* > 0.05)

**Table 4:** The variation of the ACE activity among patients according risk factors

Populations and risk factors	ACE (UI/L)		*p*
Gender	Men (n = 121)	88 ± 26.1	<0.001
	Women (n = 36)	82.4 ± 3.8	
Hypertension	Yes (n = 138)	91.8 ± 24.7	<0.00001
	No (n = 19)	49.9 ± 8.6	
Diabetes	Yes(n = 101)	86.1 ± 24.4	NS
	No (n = 56)	87.8 ± 28.2	
Tobacco	Yes(n = 98)	71.8 ± 20.1	<0.00001
	No (n = 59)	111.5 ± 27.2	
Personal cardiovascular antecedents	Yes (n = 104)	88.2 ± 26.6	NS
	No (n = 53)	83.8 ± 24.3	
Obesity	Yes(n = 82)	80.2 ± 21.4	<0.001
	No (n = 75)	93.8 ± 27.4	
Dyslipidemia	Yes(n = 63)	80.6 ± 19.7	<0.001
	No (n = 94)	90.8 ± 28.7	
Alcohol	Yes (n = 52)	85.9 ± 25.2	NS
	No (n = 105)	87.1 ± 25.9	
Sedentarity	Yes(n = 67)	87.3 ± 24.9	NS
	No (n = 90)	86.3 ± 25.6	

*NS* not significant (*p* > 0.05)

**Fig. 2:**
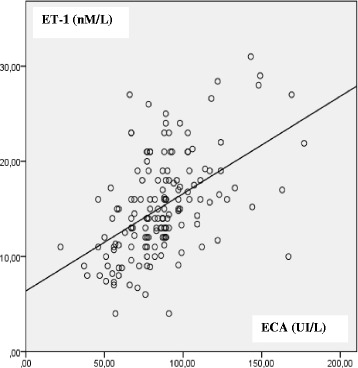
The positive correlation between ET-1 concentration and ACE activity in patients population (*r* = 0.68, *p* < 0.00001)

Besides to that, a positive statistically correlation was founded (*r* = 0.68 *p* < 0.00001) between the ET-1 concentration and ACE activity (Fig. 2).

## Discussion

Varied risk factors among patients confirm the multifactorial and polygenic origin of the ACS, well explained by the Framingham study [13, 14]. Partially balanced lipid profile in patients is caused by the diet, recommended by medical staff, by lipid-lowering therapy and in particular by the beneficial impact of the ACE inhibitors that enhance of the glucose sensitivity and the correction of lipid metabolism [14, 15].

Increased concentration of apolipoprotein B consolidates its atherogenic effects and its involvement in the genesis of ACS, although the majority of patients are under statins and anti-hypertensive medication. Unlike reversible dyslipidemia (only related to diet and lifestyle), easy to corrected by statins, mixed and genetic dyslipidemia requires a combined medication (statins + fibrates, or nicotinic acid) whereas Apo A1 (statistically higher in healthy croup) has acardio-protective effect supported by various studies. These apolipoproteins are under genetic control which showed the polygenic origin of ACS [4, 14, 16].

Furthermore, our study showed a statistically significant increase in the ET-1 values among patients compared to control subjects, which reflects its role not only in prolonged vasoconstriction but also in its atherogenic impact, its pro-oxidant effect (indirectly involved in the NADPH oxidase activation and radical oxygen species generation) and its pro-aggregating role (by his role in the thromboxane A2 synthesis). [8, 9, 16–18].

Although, 97% patients were under treatment with inhibitors of angiotensin-I converting enzyme, this enzyme activity was statistically higher among patients, reflecting the involvement of ACE as risk parameters for heart diseases. This role is well described in the literature and explained mainly by hypertensive and vasoconstrictor effects of this zinc-metallopeptidase. Recent studies also have been beginning the study of pro-oxidant role of this enzyme by activation of NADPH oxidase which triggers lipid peroxidation starting point for atherosclerosis. Actually vasoconstrictor and pro-oxidant effects accumulate in the pathophysiology of atherosclerosis and coronary syndromes [10, 11, 18]^.^

The ET-1 concentration and the ACE activity dependent, in this study on gender, hypertension, smoking,obesity and dyslipidemia, unlike diabetes, Personal cardiovascular antecedents, alcohol and physical inactivity.

The increasing concentration of ET-1 and the higher activity of the ACE in men patients is explained by hormonal and metabolic reasons (non-genetic) since they are encoded by two autosomal genes (6p24.1 for ET-1 and 17q23 for ACE), unlike the angiotensin-2 converting enzyme encoded by a gene located on the X chromosome. According to the reference Framingham study, women are protected against cardiovascular complications compared to men, this protection is manifested by blocking and inhibiting of atherogenic factors (including ET-1 and ACE) [19, 20].

The concentration and high activity in hypertensive compared with normotensive patients is explained by the vasoconstrictor effect of ET-1 and ECA and their roles in the sodium reabsorption. In hyperactivity these regulatory effects become hypertensive [9, 21, 22].

The ET-1 and the ACE inhibitions by tobacco, obesity and dyslipidemia are explained by the role of metabolic syndrome, generated by tobacco (mainly nicotine), complex lipids (among obese and dyslipidemic coronary patients). The metabolic syndrome is directly and indirectly associated with damage of the endothelial tissue, major secretory of ET-1 and ACE [23–27].

The high percentage of diabetic patients (64%) shows the impact of this risk factor in the genesis, pathophysiology and complications of ACS, manifested at different levels (oxidative and inflammation status, metabolic and thrombotic disorders…) [24].

However, diabetes was not implicated in the change of plasmatic ET-1 and ACE activity and complications of metabolic syndrome due to the used of drug and the recommended diet [24, 25].

The difference between patients with and without cardiovascular antecedents for the two parameters is not significant given the short half time life of ET-1 and ACE, drug interactions and instant regulation of these parameters (which reaches their the highest levels during the acute phase) [7, 11, 22].

Statistically positive correlation between ET-1 concentration and ECA activity is explained by the inductive effect of the angiotensin II (generated by the ACE) in the synthesis of ET-1 and thecommon activation axis of vasoconstrictors (hypovolemia, intervention ofbaro-receptors…). This activation escapes from the regulation in these pathological situations [28–31].

A multidisciplinary study that combines the interactions between enzymes and predictive peptides according the rupture and the size of the atherosclerotic plaque (measured by appropriate technical, Optical coherence tomography) can better elucidate the complex pathophysiology of ACS and more targeted the treatment [30, 32].

## Conclusion

Our study shows the impact of the ET-1 higher concentration and the ACE higher activity in the ACS predisposition and pathophysiology, seen their vasoconstrictors, pro-oxidant and pro-aggregation roles. A thorough study of each component seems important to give other therapeutic targets (ET-1 inhibitors by analogy to ACE inhibitors…). Besides to that, the study of molecular and genomics interactions between these two cardiovascular markers aim to elucidate the different metabolic cascades (activation and braking).

## Abbreviations

ACE: Angiotensin-converting enzyme
ACS: Acute coronary syndrome
Apo A: Apolipoprotein A1
Apo B: Apolipoprotein B
ET-1: Endothelin-1
SPSS: Statistical package for the sociological sciences
